# Gender and the pandemic: Associations between caregiving, working from home, personal and career outcomes for women and men

**DOI:** 10.1007/s12144-021-02630-6

**Published:** 2021-12-30

**Authors:** Vasilena Stefanova, Lynn Farrell, Ioana Latu

**Affiliations:** 1 School of Psychology, Queen’s University, 18-30 Malone Road, Belfast, BT9 5BN UK; 2 Psychology Department, National College of Ireland, Dublin, Ireland

**Keywords:** COVID-19 pandemic, Gender, Work-family conflict, Caregiving, Career aspirations

## Abstract

The coronavirus pandemic lockdowns have led to an increase of caregiving and household responsibilities for many employees while working from home. We aimed to investigate whether there was a gender imbalance in the division of household labour within families during the pandemic, and whether this imbalance was associated with gender differences in personal outcomes (work-family conflict, burnout) as well as career-related outcomes (career self-efficacy and aspirations). Participants were 240 heterosexual individuals with or without caregiving responsibilities who lived with a partner and worked from home during the pandemic. They completed self-report questionnaires and indicated the division of domestic tasks within their household, the extent to which they experienced burnout and work-family conflict, and their career aspirations and career self-efficacy. The findings showed a significant gender imbalance, such that female caregivers spent significantly less time on work compared to the other groups and significantly more time on caregiving compared to male caregivers during the lockdown. There was a significant direct effect of caregiving on career outcomes for women, such that the more caregiving women performed during the lockdown relative to other tasks, the more negative their self-reported career outcomes were. Among men, caregiving did not predict career outcomes. Overall, our study showed that the gender imbalance in distributions of caregiving duties during the pandemic is associated with negative personal and professional outcomes for women who are caregivers. Practical implications are discussed accounting for this gender imbalance in the context of the pandemic and its influence on wellbeing and career outcomes, particularly for heterosexual women.

**Supplementary Information:**

The online version contains supplementary material available at 10.1007/s12144-021-02630-6.

The advent of lockdown and social distancing measures imposed by governments across many countries during the ongoing COVID-19 pandemic has led to a conundrum for many employees: their regular work demands were expected to be met from home, however, their domestic responsibilities also increased (e.g., caregiving, home-schooling, cooking, cleaning). Employees with caregiving responsibilities also found themselves without many of the support systems and respite opportunities they relied upon before the pandemic. Many parental caregivers were left without childcare options and tasked with the further responsibility of home-schooling their children for which they felt ill-prepared (e.g., Bol, 2020; Parczewska, 2020). Caregivers for older adults (e.g., spouses or parents) had to function without crucial supports such as routine home-visits from healthcare professionals (Greenberg et al., 2020) or adult day services (Lightfoot & Moone, 2020). Many people also found themselves having to take on caregiving responsibilities during the pandemic that they did not have previously and having to balance this work with the duties of their paid employment (Chan et al., 2020). The challenge of accommodating remote work alongside household duties and full-time caregiving responsibilities meant a shift in domestic dynamics among those working from home, particularly among heterosexual couples, due to higher likelihood of traditional role distribution according to gender (male/provider and female/caregiver; Crawford, 2011). In the present study we focus on understanding whether gender moderated the distribution of daily tasks (including paid and unpaid work) during the pandemic within heterosexual couples, and whether these particular domestic dynamics were associated with differential personal and career outcomes for women vs. men. These relationships are important to understand given that they may have long-term effects for gendered outcomes in the next few years (Vaughan-Whitehead, 2011).

## The Gendered Division of Household and Caregiving Work during the Pandemic

Before the pandemic, the division of household work was largely imbalanced, with women conducting more domestic chores (e.g., McMunn et al., 2020; Treas & Lui, 2013) and contributing more to childcare (e.g., Craig & Mullan, 2011; Sandberg & Hofferth, 2001) compared to men. This pattern holds even among women who earn more than their partners (e.g., Schneider, 2011), with the suggestion that domestic responsibilities are a key source of gender performance and production (West & Zimmerman, 1987). According to Social Role Theory (Eagly & Wood, 1999; Wood & Eagly, 2012), role expectations arise from society’s division of labour by gender, specifically the assignment of domestic work and caregiving duties to women and of paid work to men. Furthermore, traditional cultural beliefs in Western society assign the role of primary caregiver to women and the role of primary breadwinner to men (Deutsch & Saxon, 1998). Cultural expectations around prescribed gender roles, can lead women and men adapting to the social roles expected of them and sets a tendency for them to devote more time to different tasks in their everyday lives (Eagly & Wood, 1999). In line with these traditional gender roles, women continue to be associated with domestic and communal roles (Wood & Eagly, 2012).

Pre-pandemic research has suggested that women, particularly mothers, face additional challenges in managing both their career and home lives (e.g., Harris & Giuffre, 2010; McIntosh et al., 2012; Meeussen & Van Laar, 2018; Peterson et al., 2018). Women’s career progression has previously been shown to be negatively impacted by career breaks and having children (e.g., McIntosh et al., 2012) with the suggestion of a motherhood penalty (e.g., Heilman & Okimoto, 2008). Alternatively, fathers may experience a fatherhood bonus in their careers with increased salary and leadership opportunities compared to men without children, for example (e.g., Hodges & Budig, 2010). As a result of the pandemic, increased domestic demands and reduced alternative support options may further compound these challenges and gendered dynamics which have affected women in particular. It was suggested that pre-existing gender inequalities have worsened since the start of the pandemic, pushing women towards a 1950’s way of living with women being the “default” parent most of the time, despite also being employed (Summers, 2020). With many countries still facing some form of lockdown restrictions including advice to work from home where possible, these domestic arrangements may continue for many heterosexual couples for the foreseeable future. It is important, therefore, to advance the literature in this domain by exploring whether a potential increase in gender imbalances in domestic labour and caregiving during lockdown is associated with negative personal and professional outcomes. The current study seeks to address this gap in the literature. If crises generally deepen existing inequalities (Vaughan-Whitehead, 2011), we may expect to see these gender imbalances in the division of unpaid, domestic labour amplified among heterosexual couples, given the specificities of the current pandemic. In the present study, we aim to explore divisions of domestic labour during the pandemic. Based on the above literature on gendered expectations based on social roles, we hypothesise that women will be likely to perform more housework and caregiving duties than men and will spend less time on paid work during the pandemic.

Early research on the pandemic seems to support this prediction, with some caveats. Petts et al. (2021) found evidence for both exacerbation and reduction of domestic gender inequalities among US parents. For example, there was a shift towards more gender equal domestic labour divisions largely driven by fathers taking on more responsibility at home than they had pre-pandemic. However, mothers were still shouldering most of the housework and childcare, and their responsibilities increased during the pandemic (Petts et al., 2021). Evidence that women completed more housework and caregiving for children and older people during the pandemic than men (despite increases in men’s domestic involvement) has been found across a number of countries (e.g., UK, Germany, the US, Adams-Prassl et al., 2020a; Australia, Craig & Churchill, 2020). Women have reported feeling more dissatisfied with how they and their partner shared domestic labour during the pandemic, with many reporting they complete more than their fair share of these tasks (Craig & Churchill, 2020).

Increased domestic duties can, in turn, impact upon time management for paid employment. During the current pandemic, mothers with young children reduced their paid work hours four to five times more than fathers to meet their increased domestic demands (Collins et al., 2020). Fathers’ work hours were less affected, even when both parents were able to work from home (Collins et al., 2020). Indeed, during the lockdown, mothers were significantly more likely to spend their work hours simultaneously trying to perform household and caregiving responsibilities, combining almost half (47%) of their paid-work time with care and household duties, compared to 30% of fathers’ paid-work time (Andrew et al., 2020). These findings suggest that parents, especially mothers, performed some childcare throughout most of their day, thus impacting on their paid employment time.

In the present study, we aim to assess the division of household labour among caregivers compared to non-caregivers and we hypothesise that caregivers will be likely to devote more time to caregiving and domestic tasks and less time to paid work, based on Social Role theory and findings showing gender imbalances in caregiving and domestic tasks. Furthermore, there is likely to be an interaction effect between gender and caregiving, as research suggests that caregiving expectations are gendered with women expected to place greater value on being a communal caregiver than men (Haines & Stroessner, 2019). For example, behaviour that suggests low prioritisation of traditional gender roles such as a neglect of caregiving duties for women, or counter-stereotypical behaviour such as engaging in more agentic paid employment, can result in backlash (e.g., Haines & Stroessner, 2019; Rudman et al., 2012). Women may, therefore, be more likely to prioritise and engage in caregiving and communal activities over breadwinning activities such as their paid employment duties. This aligns with both social role theory (Eagly & Wood, 1999) and role congruity theory (Eagly & Diekman, 2005), as well as the more recent role prioritisation model (Haines & Stroessner, 2019) which suggests that gender stereotypes contribute to cultural expectations about the degree that men and women should prioritise either work or family. In light of this, we hypothesise that female caregivers will be likely to spend more time on caregiving and less time on paid work than male caregivers, male non-caregivers and female non-caregivers during the pandemic.

## Personal and Professional Outcomes in the Context of the Pandemic

Differential distributions of domestic labour and caregiving during the pandemic are likely to contribute to relevant personal and professional outcomes. Recent research has begun to focus on describing women and men’s personal (e.g., stress, well-being) and professional (e.g., productivity) outcomes during the pandemic. The pandemic has taken its toll on personal well-being and women in particular have experienced a significant drop in mental health, increasing the gender gap (Adams-Prassl et al., 2020b; Alon et al., 2020). This trend has been supported by findings across a number of countries showing that the pandemic has had a greater psychological impact on women (Israel, Horesh et al., 2020; UK, Oreffice & Quintana-Domeque, 2021; Turkey, Özdin & Bayrak Özdin, 2020; China, Song et al., 2020). Social distancing measures and lack of typical support services have been very challenging, particularly for caregivers, with the potential to increase feelings of loneliness, isolation, stress, and burnout beyond the levels carers normally experience (Greenberg et al., 2020; Kent et al., 2020; Lightfoot & Moone, 2020). Along with increased caregiving and domestic demands, other factors associated with parental stress and burnout have been exacerbated by the pandemic, such as lack of leisure time and low levels of social support (Griffith, 2020; Hiraoka & Tomoda, 2020).

In the present study we aimed to examine the consequences of the interactive effects of gender and caregiving on personal outcomes such as work-family conflict and burnout. Previously, a meta-analysis by Michel et al. (2010) showed that family stressors and parental demands are predictors of work-family conflict. Specifically, caregivers reported more work-family conflict than non-caregivers, especially if they had more children and if their children were young (e.g., Bedeian et al., 1988; Grandey & Cropanzano, 1999). It has also been suggested that the experience of work family conflict could be associated with more negative stress-related (e.g., burnout) and career-related outcomes (e.g., lower career self-efficacy and aspirations; Allen et al., 2000). Pre-pandemic research has found that work-family conflict predicted more job dissatisfaction and parental distress, as well as lower psychological wellbeing (Kinnunen et al., 2004). Additionally, work-family conflict was positively associated with job withdrawal intentions (Greenhaus et al., 2001). In an academic context, women who experienced the tension of work-family conflict were more likely to decide to quit academia during the early stages of career development (Zuckerman, 1991). This effect was weaker for men. On the basis of this literature, we hypothesised that more time spent on household and caregiving responsibilities during the pandemic would be related to greater work-family conflict, especially among caregivers (Byron, 2005; Del Boca et al., 2020), and we aimed to examine the consequences of this conflict on career outcomes.

Regarding professional outcomes, when looking at dual-career families working from home, women self-reported lower job satisfaction and work productivity than men during the pandemic (Feng & Savani, 2020). Additionally, women’s broader career outcomes during and post-lockdown appear less promising. Across several countries such as the UK (Adams-Prassl et al., 2020a), the US (Alon et al., 2020), and Israel (Kristal & Yaish, 2020), more women have lost their jobs due to the pandemic than men. Contributing factors include the sectors that many women work in being heavily affected by lockdown measures and the demands of increased caregiving responsibilities (Alon et al., 2020). For example, scholarly productivity for academics has received a great deal of attention with early findings showing greater decreases in productivity for women and parents during the pandemic (Breuning et al., 2020; Cui et al., 2020; Myers et al., 2020). This was especially true for women with younger children and Black women; men’s productivity has been less affected (Staniscuaski et al., 2020). A consequence of this is likely to be a widening of the existing gender pay gap (Alon et al., 2020; Kristal & Yaish, 2020). Among individuals with caregiving responsibilities who were in paid employment prior to the lockdown, mothers were also significantly more likely to have been furloughed than fathers (Andrew et al., 2020). In sum, these factors could potentially produce long-term and gendered professional consequences that further increase gender inequalities in career development, especially for women with caregiving responsibilities.

## The Current Study

The current study first investigated whether there was a gender imbalance in the division of household labour among heterosexual couples during the pandemic and second, whether these labour divisions were associated with gender differences in personal (work-family and family-work conflict, burnout) and professional outcomes (career self-efficacy and aspirations) via a structural equation model that we develop below. Although gender inequalities in each of those areas have been investigated independently, our approach represents a novel step in current pandemic research—linking the division of domestic labour and caregiving with potential personal outcomes that may subsequently influence career outcomes via a SEM model involving both mediation and moderation patterns, thus offering a holistic view. Personal and professional negative outcomes are important to investigate, as they can feed into each other and create vicious cycles which may deepen gender inequalities. To illustrate, it is likely that negative personal outcomes will have a significant impact on employees’ future career outcomes as efforts to adapt to new work-life dynamics continue. The experience of burnout and stress can hinder one’s perceived self-efficacy in their work and lead to reduced career aspirations and lower achievement goals (e.g., Allen et al., 2000; Feng & Savani, 2020), which could in turn deepen gender inequalities in the workplace and, therefore, lead to differences in the performance outcomes of women and men in the long term (e.g., Hill et al., 2008). High perceived self-efficacy, career aspirations and motivation have consistently been linked to career success (e.g., Abele & Spurk, 2009). Building on this evidence, in the current study we operationalised career outcomes as including self-efficacy and career aspiration measures to assess whether the importance individuals place on their career development and their perceived capability to meet the demands of their job are impacted by work-family conflict and burnout.

In the present study we focused on heterosexual individuals with or without caregiving responsibilities (e.g., caring for children, elderly, ill relatives) during the pandemic. Considering previous literature and theory in relation to gender roles (Crawford, 2011), we predicted that women would complete more household and caregiving duties during the pandemic and may also experience lower personal and professional outcomes compared to men. The key focus, however, was whether these greater domestic demands would be associated with lower personal and professional outcomes and whether these were more significant among women. We hypothesise that the greater pressure women (particularly caregivers) experience to achieve a work-life balance that does not lessen their engagement in gender typical behaviour such as domestic work and caregiving (e.g., Haines & Stroessner, 2019) may be exacerbated during the pandemic and contribute to more negative personal and professional outcomes as women attempt to juggle their family and work roles in exceptional circumstances.

**Fig. 1:**
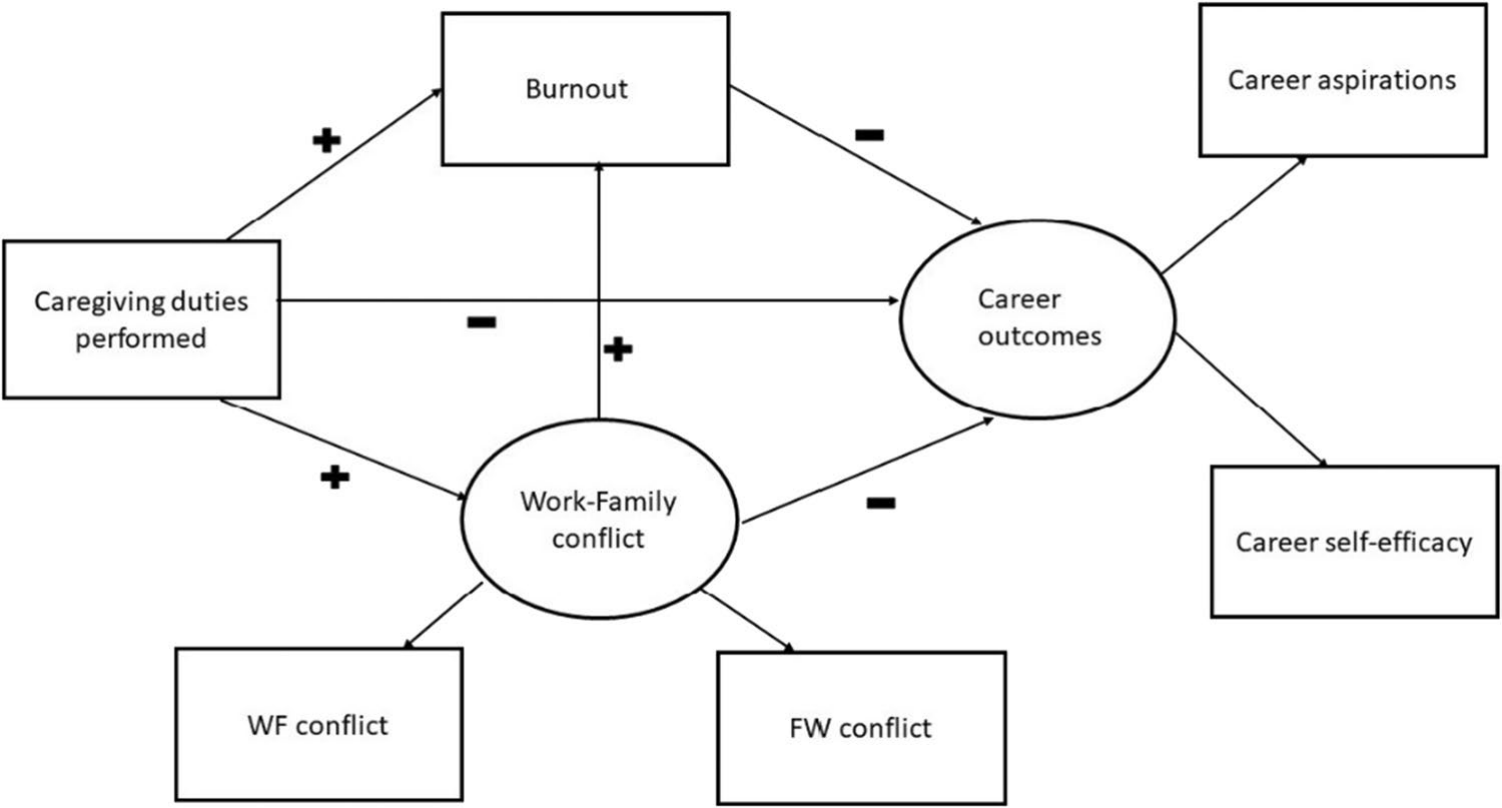
Conceptual model of the effect of caregiving duties on career outcomes. Pluses ( +) signify a predicted positive relationship and minuses (-) signify a predicted negative relationship between the variables. This model was tested for men and women separately, in a multigroup paradigm

The structural equation model built on these predictions will test the aforementioned relationships between pandemic labour distributions and personal and professional outcomes, particularly whether the proportion of caregiving and housework performed during the lockdown predicted the amount of burnout and work-family conflict experienced, as well as predicting differential career outcomes (see Fig. 1). Theoretically, this research offers the unique opportunity to understand gender differences in personal and professional outcomes in the context of a profound worldwide crisis that may have exacerbated gendered imbalances in the home. Practically, this research is important because it will generate knowledge and policy suggestions to address potential gender issues in the workplace moving forward, as we reckon with the continuation and fallout of this global pandemic. In summary, we tested the following hypotheses:

- Hypothesis 1. Women will be more likely than men to a) Perform more housework and caregiving duties during the pandemic, b) Spend less time on paid work during the pandemic, c) Report experiencing more work-family and family-work conflict during the pandemic, d) Report experiencing more burnout during the pandemic.
- Hypothesis 2. Caregivers will be more likely than non-caregivers to a) Perform more housework duties during the pandemic, b) Spend less time on paid work during the pandemic, c) Report experiencing more work-family and family-work conflict during the pandemic, d) Report experiencing more burnout during the pandemic.
- Hypothesis 3. Female caregivers will be more likely than male caregivers to a) Perform more housework and caregiving duties during the pandemic, b) Spend less time on paid work during the pandemic, c) Report experiencing more work-family and family-work conflict during the pandemic, d) Report experiencing more burnout during the pandemic.
- Hypothesis 4. More caregiving would be more likely to lead to more experiences of work-family and family-work conflict, which would lead to more burnout and, ultimately, to lower career outcomes, particularly among female caregivers.

## Method

### Participants

We invited heterosexual participants who were in a relationship, co-habiting with their partner, and were working from home during the pandemic to take part in this study. Two hundred and forty participants from a number of countries across the world completed the survey, with the majority residing in the UK or Ireland (*n* = 136), followed by the US (*n* = 31). A full list of participants’ countries of residence are included in Appendix A. One hundred and seventy-five participants were recruited via social media (e.g., Twitter, Facebook groups), fifty via Prolific and sixteen via MTurk. All participants were above 18 years of age, ranging from 18 – 66 years (*M*_*Age*_ = 34.67 years; *SD*_*Age*_ = 7.94). Almost half of the participants (*n* = 119) worked in academia in some capacity (e.g., lecturer; administrator) while the remaining 105 participants worked across a range of positions such as in the civil service or IT. Our sample consisted of 132 women and 92 men; 16 participants did not indicate their gender. Participants who did not indicate their gender were excluded from the analyses. Participants who indicated that they completed any percentage above 0 of the caregiving duties within their household during the pandemic *and* who spent any proportion above 0 of their day caregiving were categorised as having caregiving responsibilities. There were 62 women and 42 men within the sample that had caregiving responsibilities (e.g., childcare, eldercare) during the pandemic and 70 women and 50 men with no caregiving responsibilities. Participants were also asked whether their partner was working from home as well as themselves. The majority of participants’ partners were also working from home (*n* = 185; women with partners at home = 107; men with partners at home = 68), with only 55 indicating that their partners were not working from home (women with partners not at home = 25; men with partners not at home = 24).

### Measures and Materials

Participants completed the following measures to assess their eligibility, time spent completing domestic and professional tasks, division of domestic labour, and relevant personal and professional outcomes.

#### Eligibility Check

First, participants were asked to indicate if they worked from home as a result of the pandemic, if they were in a heterosexual relationship and if they were co-habiting with their partner. If they responded with ‘yes’ to these three questions, they were eligible to participate in the study and proceeded to the remaining questionnaires below. If they responded with ‘no’ to these initial questions, they were referred to the Debrief directly.

#### Distribution of Duties at Home within the Household During the Pandemic

Participants were asked to indicate from 0–100% the extent to which different members of their household (self, partner, others) contributed to caregiving and housework during the pandemic.

#### Proportion of Day Spent on Daily Activities

Participants were asked to indicate what proportion of their day (from 0–100%) they spent on a number of daily activities including paid work, housework and caregiving during the pandemic and before the pandemic.

#### Work-family and Family-Work Conflict Scales (adapted from Netemeyer et al., 1996).

Participants completed two scales, each consisting of five items, that assessed whether balancing work and family responsibilities created inter-role conflict for them. The scales were adapted from Netemeyer et al. (1996) by having participants respond on a 5-point scale instead of a 7-point scale. Participants indicated their agreement or disagreement with the items on a 5-point scale which ranged from 1 ‘*Strongly Disagree*’ to 5 ‘*Strongly Agree*’. Five items assessed work-family conflict whereby the demands of work interfered with familial duties (e.g., ‘My work has a negative impact on my family life’; *M* = 3.02, *SD* = 0.9, α = 0.84). The other five items assessed family-work conflict whereby the demands of family interfered with work-related duties (e.g., ‘My work performance suffers because of my personal and family commitments’; *M* = 2.6, *SD* = 0.98, α = 0.86). Higher scores indicated greater work-family and family-work conflict, respectively.

#### Burnout Measure—Short Version (Malach-Pines, 2005)

This measure consisted of 10 items and assessed burnout among participants in relation to their work. Burnout is defined here as perceived emotional, physical, and mental exhaustion. Participants indicated on a 5-point scale from 1 ‘*Never’* to 5 ‘*Always’* how often they felt negatively about their paid work (e.g., ‘hopeless’, ‘depressed’, ‘disappointed with people’). Higher scores indicated higher levels of burnout (*M* = 2.47, *SD* = 0.69, α = 0.87).

#### Career Aspirations Measure (adapted from the Leadership aspirations scale, Simon & Hoyt, 2013, and the Leadership identification scale, Hoyt & Blascovich, 2007)

Participants rated 8 items on a 5-point scale, from 1 ‘*Strongly disagree*’ to 5 ‘*Strongly agree*’. This scale involved items that were taken from two different scales – Simon and Hoyt (2013) Leadership aspirations scale and Hoyt and Blascovich’s (2007) Leadership identification scale. The items were adapted to measure participants’ career motivation instead of leadership motivation (e.g. the word ‘leadership’ was replaced with the word ‘career’ where questions such as ‘I am a leadership-oriented person’ became ‘I am a career-oriented person’). The 2 items taken from the Leadership aspirations scale assessed participants’ future career aspirations (e.g., ‘I will actively do things to advance my career in the future’), reflecting the importance one places on their future career progression, as well as one’s motivation and confidence to advance in their career. The 6 items taken from the Leadership identification scale assessed the extent to which one saw oneself as a career leader (e.g. ‘My career is important to me’). Higher scores indicated more positive career aspirations (*M* = 3.91, *SD* = 0.67, α = 0.874).

#### Career Self-Efficacy Measure (adapted from the Self-efficacy for Leadership scale, Murphy, 1992)

Participants responded to 4 statements assessing their belief in their own ability to pursue their career goals (e.g., ‘I am confident of my ability to achieve what I want to accomplish in my career’). The scale was adapted, where items were rated on a 5-point scale ranging from 1 ‘*Strongly disagree*’ to 5 ‘*Strongly agree*’, instead of participants responding with either Yes or No as in the original scale. Self-efficacy in the context of this measure is defined as the perception of one’s own career capabilities and was previously reported to predict motivation for goal attainment in a career setting (e.g., Murphy, 1992). Higher scores indicated greater career self-efficacy (*M* = 3.76, *SD* = 0.68, α = 0.808).

#### Demographics

Finally, participants were asked to indicate their gender, parental status, level of education, profession, age, nationality, and country of residence.

### Procedure

Data collection ran from May 15^th^ to July 3^rd^ 2020, thus beginning approximately two months after authorities in the UK, US and in most European countries announced the initial major lockdown restrictions including school closures and work from home requirements. The majority of participants were initially recruited via social media by posting a survey link (hosted on Qualtrics) on Twitter and Facebook. Personal contacts of the authors were also asked to share the survey with their colleagues and acquaintances on social media. Additionally, a number of eligible participants were recruited from MTurk and Prolific and received $1 and £1 respectively for their participation. Participants completed an online survey consisting of the measures detailed above which assessed their domestic and professional responsibilities during the pandemic, the division of labour within their household, experiences of work-family and family-work conflict (Netemeyer et al., 1996), burnout (Malach-Pines, 2005), and career self-efficacy and aspirations, as well as demographic variables. After completing the survey, participants were debriefed and thanked.

## Results

### Common Method Variance

In addition to incorporating procedural remedies to reduce the impact of common method variance within the study (e.g., implementing anonymous responding, and different scale endpoints and formats for the predictor and criterion measures; Podsakoff et al., 2003), a statistical exploration was conducted. In order to assess whether common method variance impacted the data, we conducted Harman’s Single-Factor Test, which is widely used in the literature (Podsakoff et al., 2003). We examined the unrotated factor solution to determine the number of factors that were necessary to account for the variance among the variables. The findings showed the presence of six distinct factors with eigenvalues greater than 1.00, rather than a single factor. As no single factor emerged from this analysis, this suggests that there was not a substantial amount of common method variance present in this study.

### Analytic Plan

Our first hypothesis focused on potential gender imbalances in time allocation. For the purposes of this study, we focused on the effects of gender and caregiving *during* the lockdown. However, comparisons of time spent on daily tasks did show that caregivers spent significantly less time on work and more time on caregiving during the pandemic than before (see Appendix B for full data and analyses of comparisons before vs during the pandemic). We first assessed whether the proportion of time per day spent on work and household tasks during the lockdown differed based on participants’ gender and caregiver status by conducting four 2 × 2 between-subjects ANOVAs where the dependent variables were proportion (%) of time per day spent on work and housework. Next, we conducted a one-way ANOVA to assess whether the proportion (%) of time per day spent on caregiving differed based on the gender of participants who are caregivers.

Additionally, we conducted independent sample t-tests to compare female and male participants’ estimations of the proportion (%) of the overall caregiving and housework duties that they themselves completed during lockdown. We also conducted four paired sample t-tests to compare the proportion (%) of caregiving and housework duties that participants reported that they performed compared to what they estimated their partners performed.

To answer our focal question as to whether these domestic labour distributions are associated with differential personal and professional outcomes for women vs. men, we conducted two related analyses. First, we conducted five 2 × 2 between-subjects ANOVAs to assess whether participants’ gender and caregiver status are associated with the degree of experienced burnout, work-family and family-work conflict, career aspirations and career self-efficacy during the lockdown. Second, we focused on the data obtained from participants who are caregivers and conducted multigroup structural equation modelling analyses (SEM) to assess whether caregiving duties and housework load predicted burnout and work-family/family-work conflict, in turn influencing career aspirations and self-efficacy for women and men separately. As our conceptual model in Fig. 1 shows, work-family conflict was a latent variable consisting of work-family conflict and family-work conflict. Similarly, career outcomes was a latent variable consisting of career aspirations and career self-efficacy. We used the model to test our mediation hypothesis that there will be a significant positive relationship between caregiving and work-family conflict, as well as between work-family conflict and burnout, and a negative relationship between burnout and career outcomes. Additionally, we hypothesised a negative direct relationship between caregiving and career outcomes.

## Gendered Distribution of Time and Domestic Duties

### Distribution of time spent on daily activities

**Fig. 2:**
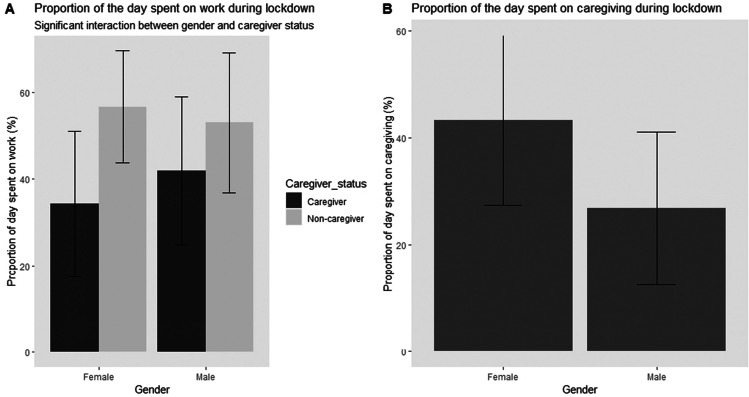
Proportion of the day spent on paid work (panel **a**) and caregiving (panel **b**) during the lockdown

We aimed to assess whether the distribution of time allocated for different activities during the lockdown (work, caregiving, housework) differed by gender and caregiver status. We conducted two 2 (Gender: Male vs Female) × 2 (Caregiver status: Caregiver vs Non-caregiver) ANOVAs on the proportion (%) of time per day spent on work and housework. Table 1 contains a summary of the results. The findings showed a main effect of caregiver status, where individuals with caregiving responsibilities spent less time on work (*M* = 37.34, *M*difference = 17.76) during the lockdown than individuals without caregiving responsibilities, providing support for Hypothesis 2b This was qualified by a significant interaction between gender and caregiver status for proportion of time spent on work. Planned contrasts were conducted to follow-up on this significant interaction between gender and caregiver status and the findings demonstrated that female caregivers (*M* = 34.31%, *SD* = 16.75) spent significantly less time on work compared to female non-caregivers (*M* = 56.67%, *SD* = 12.84), *t*(220) = 8.27, *p* < 0.001, Cohen’s *d* = 1.49, while male caregivers (*M* = 41.95%, *SD* = 17.12) spent significantly less time on work compared to male non-caregivers (*M* = 52.90%, *SD* = 16.17), *t*(220) = 3.36, *p* < 0.001, Cohen’s *d* = 0.66. Results also suggested that female caregivers spent significantly less time per day on paid work (*M* = 34.31%, *SD* = 16.75) than male caregivers (*M* = 41.95%, *SD* = 17.12), *t*(220) = 2.49, *p* = 0.01, Cohen’s *d* = 0.45. Therefore, women who are caregivers spent significantly less time completing their paid work duties during the lockdown compared to the other participant groups (see Fig. 2, panel a), supporting Hypothesis 3b.

**Table 1:** Results from the 2 (Gender: men or women) × 2 (Caregiver status: caregiver or non-caregiver) ANOVAs on time allocation outcomes (work and housework). Significant results are highlighted in bold

Independent variable effect	Outcome	*N*	*F*	df	*p*	Cohen’s *d*
Gender	Time spent on work	224	.88	220	.35	0.10
Caregiver status	**Time spent on work**	224	**62.07**	**220**	** < .001**	**1.12**
Gender x Caregiver status	**Time spent on work**	224	**7.37**	220	.007	
Gender	Time spent on housework	224	.58	220	.45	0.10
Caregiver status	Time spent on housework	224	1.06	220	.30	0.20
Gender x Caregiver status	Time spent on housework	224	.36	220	.55

Additionally, we conducted a one-way ANOVA to assess the effect of gender (Male vs Female) on proportion of time (%) per day spent on caregiving. Only the data from participants who were caregivers were included in this analysis. The findings showed that women spent significantly more time on caregiving (*M* = 43.24%, *SD* = 15.89) than men (*M* = 26.79%, *SD* = 14.24), *F*(1, 103) = 29.16, *p* < 0.001, Cohen’s *d* = 1.09 (see Fig. 2, panel b). This finding supports Hypothesis 1a and 3a.

Overall, consistent with our hypotheses, these findings show a gender imbalance in time spent on daily activities and household duties during the lockdown, with women performing more caregiving than men. Additionally, caregivers were impacted more negatively by the lockdown than non-caregivers in terms of the ability to dedicate time to perform paid work duties, especially female caregivers.

## Domestic Duties Performed by Self Compared to Partner

### Caregiving

**Fig. 3:**
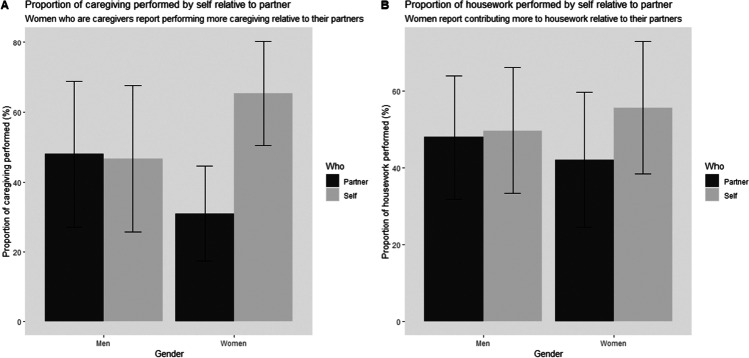
Proportion of caregiving (panel **a**) and housework (panel **b**) performed by the participants themselves compared to their partners

This analysis was conducted only on data from participants identified as caregivers (*n* = 104) to determine whether women performed more caregiving duties than their partners during the lockdown. We first compared female to male participants’ estimations of the proportion of caregiving duties that they performed and found that women reported contributing significantly more to caregiving during the lockdown (*M* = 65.37%, *SD* = 14.89) compared to men (*M* = 46.6%, *SD* = 21.05), *t*(102) = 5.33, *p* < 0.001, Cohen’s *d* = 1.03. Furthermore, women completed significantly more caregiving than their partner, *t*(61) = 10.6, *p* < 0.001, Cohen’s *d* = 2.42, while men’s self-reported share of caregiving did not significantly differ from their partner’s, *t*(41) = 0.23, *p* = 0.82, Cohen’s *d* = 0.06 (see Fig. 3, panel a).

### Housework

Again, we compared female to male participants’ estimations, and found that women reported contributing more to housework during the lockdown (*M* = 55.61%, *SD* = 17.18) compared to men (*M* = 49.66%, *SD* = 16.3), *t*(222) = 2.6, *p* = 0.01, Cohen’s *d* = 0.35. We then assessed female participants’ estimations of their own contributions to housework compared to their partner’s contribution. We found that women reported contributing more to housework (*M* = 55.61%, *SD* = 17.18) compared to their male partner (M = 42.02%, SD = 17.57), and this difference was significant and large, *t*(131) = 4.61, *p* < 0.001, Cohen’s *d* = 0.78. Similar to caregiving, men’s estimation of their contribution to housework during the lockdown (*M* = 49.66%, *SD* = 16.30) was not significantly different from their estimation of the contribution of their female partner (*M* = 47.89%, *SD* = 16.02), *t*(91) = 0.54, *p* = 0.59, Cohen’s *d* = 0.11, (see Fig. 3, panel b). These findings point to disparities between women and their male partners in terms of estimated share of caregiving and housework duties that they are performing.

## Gendered Outcomes

### Personal Outcomes

**Fig. 4:**
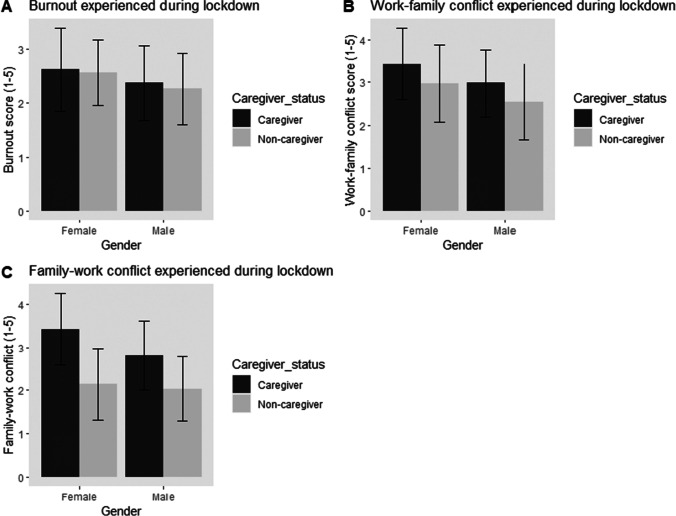
Burnout (panel **a**), Work-family (panel **b**) and Family-work conflict (panel **c**) experienced by participants during lockdown

We also aimed to assess whether the burnout, work-family and family-work conflict that participants experienced during the lockdown differed based on their gender and caregiver status. We conducted 2 (Gender: Male vs Female) × 2 (Caregiver status: Caregiver vs Non-caregiver) ANOVAs on the following outcomes: burnout scores, work-family and family-work conflict scores (see Table 2). A consistent significant main effect of gender was found, where women experienced more burnout (*M* = 2.58, *M*difference = 0.27), work-family conflict (*M* = 3.19, *M*difference = 0.44) and family-work conflict (*M* = 2.74, *M*difference = 0.36) compared to men (see Fig. 4, panels a and b). These findings provide support for Hypotheses 1c and 1d.

**Table 2:** Results from the 2 (Gender: men or women) × 2 (Caregiver status: caregiver or non-caregiver) ANOVAs on burnout, work-family conflict, family-work conflict, career self-efficacy and career aspirations. Significant results are highlighted in bold

Independent variable effect	Outcome	*N*	*F*	df	*p*	*Cohen’s d*
Gender	**Burnout**	**223**	**8.43**	**219**	**.004**	**0.40**
Caregiver status	Burnout	223	.88	219	.35	0.10
Gender x Caregiver status	Burnout	223	.08	219	.78	
Gender	**Work-family conflict**	**224**	**14.16**	**220**	** < .001**	**0.50**
Caregiver status	**Work-family conflict**	**224**	**14.59**	**220**	** < .001**	**0.52**
Gender x Caregiver status	**Family-work conflict**	**224**	.15	220	.90	
Gender	**Family-work conflict**	**224**	**10.69**	**220**	**.001**	**0.37**
Caregiver status	**Family-work conflict**	**224**	**86.76**	**220**	**.007**	**1.29**
Gender x Caregiver status	**Family-work conflict**	**224**	**5.09**	**220**	**.025**	
Gender	**Career aspirations**	**223**	**13.64**	**219**	** < .001**	**0.51**
Caregiver status	Career aspirations	223	0.13	219	.72	0.10
Gender x Caregiver status	Career aspirations	223	1.32	219	.25	
Gender	Career self-efficacy	223	2.51	219	.12	0.23
Caregiver status	Career self-efficacy	223	.33	219	.57	0.08
Gender x Caregiver status	Career self-efficacy	223	.01	219	.93

A significant main effect of caregiver status was also found, where caregivers experienced more work-family (*M* = 3.25, *M*difference = 0.45) and family-work conflict (*M* = 3.17, *M*difference = 1.07), providing support for Hypothesis 2c This was qualified by a significant interaction between gender and caregiver status for family-work conflict experienced during the lockdown. Planned contrasts were conducted to follow-up on this significant interaction between gender and caregiver status and the findings showed that women who are caregivers (*M* = 3.4, *SD* = 0.83) experienced significantly more family-work conflict compared to women who are not caregivers (*M* = 2.14, *SD* = 0.83), *t*(220) = 9.27, *p* < 0.001, and men who are caregivers (*M* = 2.8, *SD* = 0.80) experienced significantly more family-work conflict compared to men who are not caregivers (*M* = 2.03, *SD* = 0.74), *t*(220) = 4.58, *p* < 0.001. Female caregivers (*M* = 3.4, *SD* = 0.83) also experienced significantly more family-work conflict compared to male caregivers (*M* = 2.8, *SD* = 0.80), *t*(220) = 3.74, *p* < 0.001, suggesting that female caregivers experienced significantly more family-work conflict compared to the other groups (see Fig. 4, panel c). These findings provided support for Hypothesis 3c. Overall, consistent with our predictions, the lockdown induced more negative personal psychological outcomes in women compared to men. Additionally, caregivers experienced more conflict between work and family during the lockdown compared to non-caregivers, especially female caregivers.

#### Professional Outcomes

We conducted 2 (Gender: Male vs Female) × 2 (Caregiver status: Caregiver vs Non-caregiver) ANOVAs on the following outcomes: career aspirations and career self-efficacy. The findings showed a main effect of gender on career aspirations, with women having higher career aspirations (*M* = 4.04, *SD* = 0.62) than men (*M* = 3.7, *SD* = 0.7). However, all other effects regarding career aspirations and career self-efficacy were non-significant (see Table 2).

## Predicting Personal and Professional Outcomes

Our initial analyses indicated some domestic inequalities between men and women as well as caregivers and non-caregivers during the pandemic. We investigated whether these inequalities predicted different personal and professional outcomes for women and men. We used structural equation modelling (SEM) in AMOS (Arbuckle, 2014) to develop and test models that assessed the impact of caregiving duties performed relative to other daily activities on burnout and work-family conflict (personal outcomes) and career aspirations and self-efficacy (professional outcomes). We used multigroup SEM to assess these paths for male and female caregivers separately. Figure 1 presents the conceptual model examined whereby we predicted that more caregiving duties would lead to more work-family conflict and burnout resulting in negative career outcomes (i.e., lower career aspirations and self-efficacy). Work-family conflict was included in the model as a latent variable consisting of participants’ work-family conflict score and family-work conflict score. Career outcomes was also a latent variable consisting of the career aspirations score and career self-efficacy score. The cut-off criteria for the evaluation of the model involved a nonsignificant chi-square value (Kline, 2015), comparative fit index (CFI) > 0.95, root mean square error of approximation (RMSEA) < 0.08, and standardised root mean squared residual (SRMR, Hu & Bentler, 1999) < 0.08.

Correlations and descriptive statistics were calculated using IBM SPSS Statistics (version 26) and are displayed in Table 3. The levels of skewness and kurtosis were acceptable between ± 1. We tested the model by comparing it to the saturated model and, in accordance with common procedure for model development (e.g. Stevenson et al., 2020), we retested it excluding nonsignificant paths. The fit statistics indicated a good model fit for our final model, χ2(14) = 21.53, *p* = 0.09, RMSEA = 0.07, adjusted goodness of fit index (AGFI) = 0.82, CFI = 0.95, SRMR = 0.08, Akaike’s information criterion (AIC) = 77.53. Bootstrapping was performed at 95% CI to provide a robust test of our model.

**Table 3:** Descriptive statistics and correlation analyses

Variable	*N*	Min	Max	*Mean*	*SD*	Skewness	Kurtosis	1	2	3	4	5
1 Work-family conflict	108	1.00	5.00	3.27	85	05	36					
2 Family-work conflict	108	1.00	5.00	3.18	.85	-17	-.45	41**				
3 Career aspirations scale	108	1.50	5.00	3.89	.63	-.45	.9	.25**	-.02			
4 Career self- efficacy scale	109	1.75	5.00	3.79	67	-.16	-.11	.15	-.02	.66**		
5 Caregiving (%)	109	5.00	80.00	37.1	17.09	.17	-.42	.2*	.33**	-.11	-.17	
6 Burnout	109	1.10	4.50	2.51	.74	.37	-.27	.57**	.4**	-.05	-.09	.13

**Fig. 5:**
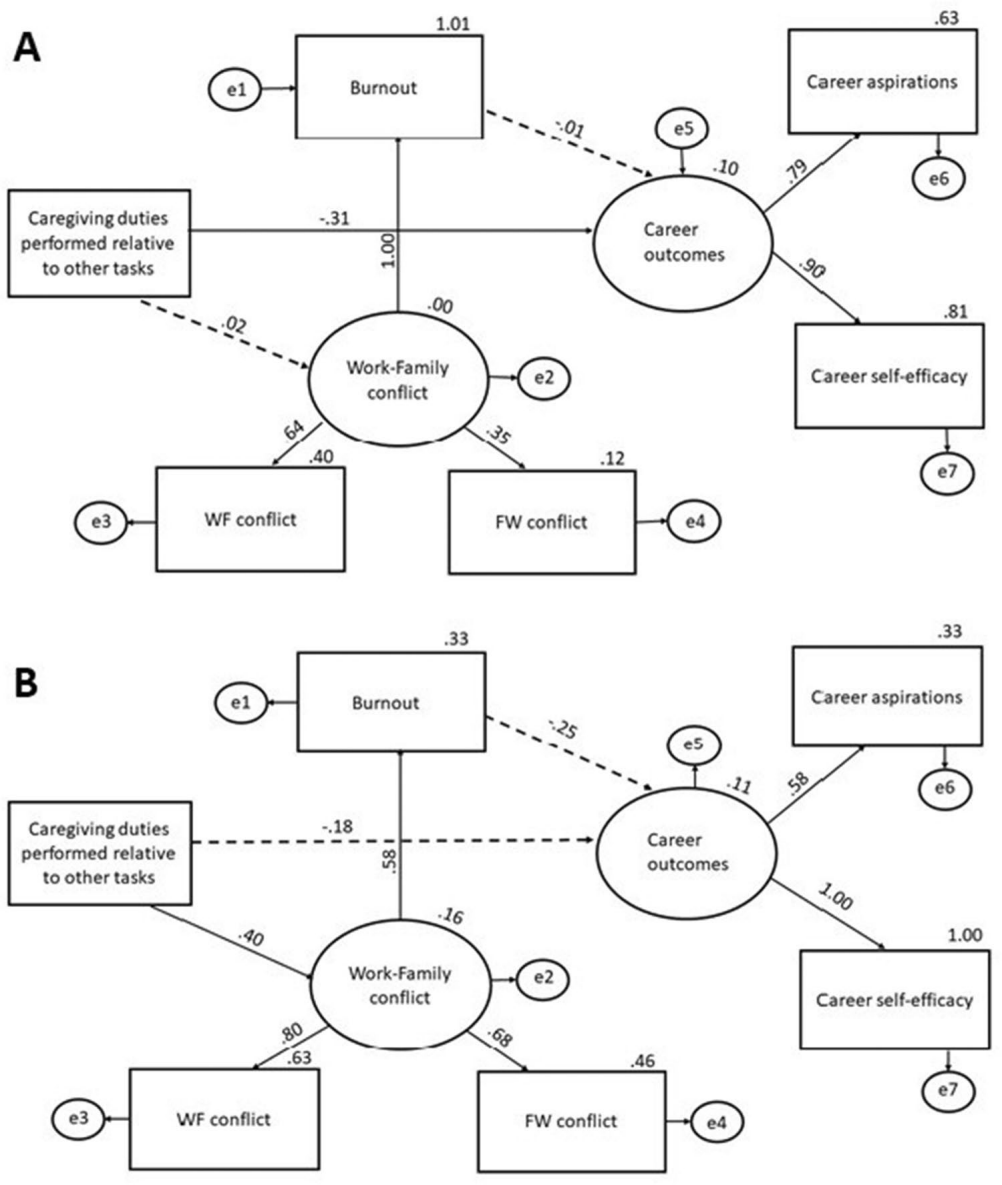
Structural model representation for female caregivers (panel **a**) and male caregivers (panel **b**). Solid lines represent significant paths and dotted lines represent non-significant paths

Consistent with the multigroup SEM recommendations, in order to assess the moderating role of gender, we constrained the paths in the female caregivers’ model and the male caregivers’ model to equality and compared the fit of the constrained model to that of the unconstrained one. The findings showed a significant difference between the constrained and the unconstrained models, *χ*^*2*^(7) = 15.22, p = 0.03, suggesting that the moderating effect of gender is significant, and that the female caregivers’ model and male caregivers’ model differ significantly. The findings indicated a significant direct effect of caregiving on career outcomes for women, where the more caregiving women performed during the lockdown relative to other daily activities, the more negative career outcomes they had, *b* = -0.31, 95% CI [-0.54, -0.02], *β* = -0.009, *p* = 0.03 (see Fig. 5, panel a). Among men, a significant positive relationship between caregiving and work-family conflict was found, *b* = 0.4, 95% CI [-0.14, 0.67], *β* = 0.02, *p* = 0.025. However, caregiving did not significantly predict men’s career outcomes (see Fig. 5, panel b). Finally, experiences of greater work-family conflict led to more burnout for both women (*b* = 1.01, 95% CI [0.69, 2.2], *β* = 1.46, *p* = 0.002) and men (*b* = 0.58, 95% CI [0.21, 0.97], *β* = 0.64, *p* = 0.011), although the effect was greater for women.

Overall, our findings show that more caregiving performed during the lockdown directly leads to more negative career outcomes for women who are caregivers. This finding provides partial support for Hypothesis 4 and our initial model predictions, where female caregivers experienced a direct impact of caregiving on career outcomes but the paths between caregiving and work-family conflict, as well as from burnout to career outcomes, were non-significant. For male caregivers, although caregiving did predict more work-family conflict this was not found to have an impact on career outcomes.

We also developed and tested a model that similarly assessed the impact of housework duties performed by male and female participants during the pandemic on career outcomes. The fit statistics indicated a good fit for the model, χ2(14) = 12.78, *p* = 0.54, RMSEA < 0.001, CFI = 1.00, SRMR = 0.05, Akaike’s information criterion (AIC) = 92.78. However, there were no significant effects of housework on professional outcomes for women and men.

## Discussion

The aim of the present study was to assess whether there was a gender imbalance in the division of household duties among heterosexual couples during the pandemic lockdown, as well as to investigate whether these domestic labour divisions predicted gender differences in personal (work-family and family-work conflict, burnout) and professional outcomes (career self-efficacy and aspirations). The findings partially supported our predictions, such that women were found to perform more caregiving and spent less time on paid work duties during the lockdown compared to men. Additionally, during the initial lockdown women experienced more burnout, work-family and family-work conflict compared to men. Caregivers were found to experience more conflict between work and family compared to non-caregivers and were less able to spend time on paid work duties during the lockdown compared to non-caregivers, and this was especially the case for female caregivers. These findings are consistent with previous research that focused on describing gender imbalances during the pandemic (e.g. Collins et al., 2020; Craig & Churchill, 2020), and suggest that women, especially those who are caregivers, might be more negatively impacted in these domains by the lockdown compared to men.

However, our prediction that gender and caregiving status would impact on career outcomes was only partially supported by our results. Interestingly, women actually reported higher career aspirations than men which might not have been expected given their more negative personal outcomes. It may be that, despite experiencing more burnout and work-family conflict, women’s motivation has not yet been affected by the pandemic. This finding may also possibly indicate that women have developed resilience over time as they have juggled work and family responsibilities long before the pandemic (e.g., Ezzedeen & Ritchey, 2009) while having to maintain high levels of career motivations to succeed. Previous research, for example, has similarly found that women reported higher career aspirations compared to men despite women also demonstrating higher perceptions of carrier barriers including multiple-role conflict, and conflict between career and family demands (Watts et al., 2015).

We were also interested in exploring the relationship between caregiving, and personal and professional outcomes and aimed to assess whether an imbalance in the distribution of caregiving duties predicted career outcomes of male and female caregivers through the burnout and work-family conflict they experienced. The findings of our SEM analysis showed that the career aspirations and self-efficacy of female caregivers were directly impacted by the proportion of caregiving they performed, such that the more caregiving they performed relative to other daily activities, the lower or more negative career outcomes they had. This effect was not observed for male caregivers. One potential reason why caregiving impacted women’s career outcomes more than men could be related to traditional gender roles. Caregiving is not traditionally a part of male gender roles but often comprises a core element of women’s (e.g. Deutsch & Saxon, 1998). Thus, there may be lower expectations for men to perform caregiving duties even under exceptional circumstances. Women on the other hand are typically expected to prioritise caregiving and domestic duties and this has likely been amplified by the current pandemic which has condensed work and home lives into the same space for many. This, therefore, could lead to more experiences of work-family conflict and negative career outcomes for women compared to men.

Contrary to our predictions, the relationship between caregiving and career outcomes was not mediated by work-family conflict and burnout. One potential explanation for this could be that, as suggested above, women’s work-family conflict and burnout are not directly related to their career motivations due to historically having to juggle family and work responsibilities while demonstrating high career aspirations should they wish to remain and advance in the paid workforce (e.g., Ezzedeen & Ritchey, 2009; Whitmarsh et al., 2007).

## Theoretical Implications

The findings of our study have valuable theoretical implications as they suggest that gender imbalances deepen during a crisis, consistent with Vaughan-Whitehead’s (2011) analysis. These imbalances appeared to follow stereotypical patterns for the most part with female caregivers, in particular, performing more caregiving and spending less time on paid work during lockdown. Our findings are also consistent with social role theory (Eagly & Wood, 1999) and role prioritisation model (Haines & Stroessner, 2019) as women were shown to devote more time to caregiving than men in the context of the pandemic. This may reflect cultural expectations that women should place greater value on these caregiving duties. Additionally, the findings of our study are novel in showing that these gender imbalances, specifically in regard to the performance of caregiving duties, can predict career outcomes. Consequently, our findings show that the significant gender imbalance in the provision of caregiving during lockdown can be associated with lower career aspirations and career self-efficacy among women. This may negatively impact the career-related choices women subsequently make. Previous research has demonstrated that women’s career self-efficacy and career advancement goals at career entry impact their salary and status in the workplace after three years (Abele & Spurk, 2009) and can also affect their likelihood to persist in a job search, as well as their interest in career exploration and development (Sterrett, 1998; van Ryn & Vinokur, 1992). This finding suggests that lower career aspirations and self-efficacy could negatively predict career development in the long term.

Additionally, female caregivers’ reduced working hours during the pandemic may have an impact on employers’ attitudes towards these women and their career opportunities and may strengthen the stereotype that ‘mothers work less’ (e.g. Heilman & Okimoto, 2008). This could, in turn, lead to assumptions that female caregivers are less committed to their jobs and may consequently limit their career opportunities post-pandemic, for example through a reduced likelihood to be recommended for promotion (e.g. Heilman & Okimoto, 2008; King, 2008). This reduction in working hours due to the pandemic may also have negative career impacts similar to the negative consequences that can accompany career breaks necessitated by motherhood (McIntosh et al., 2012).

## Practice Implications

These findings also have important practical implications, specifically for generating policy recommendations in the workplace. The gender imbalance in household duties for couples working from home during the lockdown could be taken into account by employers to inform hiring and promotion practices. This is particularly salient for caregiving. The reduced career aspirations and self-efficacy that women with caregiving responsibilities experience due to increased caregiving during lockdown could further widen the gender gap in workplaces. Therefore, more family-friendly policies could help alleviate the effects of this gender imbalance in the workplace, allowing for flexible working arrangements and augmented work expectations for caregivers that would not be detrimental to their career trajectory should they apply for a promotion or for a new professional role.

We need gender-aware policies that value domestic, unpaid work (Bahn et al., 2020), and more robustly acknowledge and accommodate childcare and caregiving arrangements. This will be necessary also when considering further lockdown restriction plans. More flexible working arrangements could be advocated for women with caregiving responsibilities during lockdown which could help alleviate the negative impact of caregiving on career progression and improve work-life balance.

## Limitations and Future Directions

A limitation of the present study is that we did not collect data from couples, making a dyadic analysis impossible. Also, due to the subjective nature of our estimation measures, there is a possibility that participants may have over- or underestimated the household duties that they themselves and/or their partners contributed to. It would have been informative to assess and compare the reports of household duties performed by partners more directly or in an objective way via observational studies, for example. However, given the restraints of the pandemic an online survey was our most feasible approach. The present study also used a convenience participant sample from different countries and this data collection approach did not allow us to explore between-country comparisons due to the country samples not being representative of their respective populations.

Additionally, although we wanted to obtain an initial snapshot of gender imbalances in the context of the pandemic in its first months, we acknowledge that collecting longitudinal data at different stages during the pandemic would be beneficial. Longitudinal studies will allow researchers to monitor whether psychological and career outcomes vary over time and whether these variations coincide with reductions in caregiving duties due to the reopening of schools and day-care centres. Longitudinal work will also be able to establish whether gender imbalances, personal outcomes, and career outcomes feed into each other creating vicious circles that deepen existing inequalities.

Future research could explore not just how gender and caregiving impact career outcomes but also how other characteristics may interact and have an impact, such as socioeconomic status (SES). For example, more stable jobs and a steady income during the lockdown is highly likely to produce different personal and professional outcomes compared to jobs which involve more insecurity. Previous research by Emslie & Hunt (2009) revealed that SES is an important factor to consider when assessing issues with work-life balance in that it impacts how women manage different roles in their lives. For example, for women at low SES, limited resources and a lack of flexibility may cause more issues with work-family conflict, while at mid- to high SES where women have more resources to employ help and have more freedom to reduce their working hours to improve their work-life balance, experiences of work-family conflict may be alleviated. Race/ethnicity is also important to consider given the disparities already being highlighted for Black, Asian and minority ethnic (BAME) individuals during the COVID-19 pandemic (Hu, 2020). Overall, future work should investigate individuals who are at the intersection of multiple marginalised identities in the workplace (e.g., race/ethnicity, SES), as they may be even more vulnerable in the face of the pandemic, being disproportionately employed in jobs that cannot accommodate work-from-home arrangements (Ali et al., 2020). Additionally, future research could focus on between-country comparisons in exploring the impact of the pandemic on women and men who are caregivers to determine whether there are cross-cultural differences perhaps influenced by variations in gender roles and expectations (Wood & Eagly, 2012). This would be helpful in assessing the extent to which potential gender imbalances in caregiving and housework duties during the pandemic are area-specific and culture-specific.

## Conclusions

Our findings provide initial evidence about how lockdown can contribute to potentially long-term gendered outcomes in career development and personal wellbeing, which offers a basis for further exploration of the shift in domestic dynamics in times of crisis. Overall, the present study suggested that there was a gender imbalance in the division of household labour during lockdown, such that women completed more caregiving and spent less time on work compared to men. Additionally, women, especially female caregivers, experienced more burnout and work-family conflict compared to men. Caregiving during the pandemic in particular had a direct negative impact on women’s self-reported professional outcomes. Lower career aspirations and career self-efficacy among women may negatively impact their career-related choices (e.g., Abele & Spurk, 2009). Gender-aware policies recognising the additional demands of domestic duties, particularly on women and caregivers, are necessary both in the workplace and in response to further lockdown restrictions to help mitigate the potential negative impact of increases in these duties on personal and professional outcomes.

## Supplementary Information

Below is the link to the electronic supplementary material.

Supplementary file1 (DOCX 15 KB)

## Data Availability

The datasets analysed during the current study are available from the corresponding author on reasonable request.
