# Supplementary material for: Gender and the pandemic: Associations between caregiving, working from home, personal and career outcomes for women and men
Source: Curr Psychol. 2021 Dec 30:1–17. Online ahead of print. doi: 10.1007/s12144-021-02630-6 (PMC8717695; doi:10.1007/s12144-021-02630-6)
Supplement: Supplementary file 1 — Supplementary file1 (DOCX 15 KB) [file 12144_2021_2630_MOESM1_ESM.docx]

**Gender and the pandemic: Associations between caregiving, working from home, personal and career outcomes for women and men**

**Supplementary Information**

**Appendix A. Participants’ Countries of Residence**

| **Country of residence Number of participants** |
| --- |
| United Kingdom 117  Ireland 19  United States 31  Spain 7  Portugal 8  Poland 9  The Netherlands 7  Italy 4  Germany 3  Canada 2  Belgium 2  Switzerland 2  Australia 2  Bulgaria 1  Romania 1  Japan 1  Greece 1  India 1  New Zealand 1  Denmark 1  South Africa 1  Sweden 1  Austria 1  Did not indicate their country of residence 17   \|  \| \| --- \| |

**Appendix B**. **Time Spent on Tasks Before vs During the Pandemic**

We aimed to assess whether the proportion of time spent on different tasks (e.g. work, caregiving, hobbies, exercise) during the lockdown has changed from before the pandemic, and whether this depended on individuals’ gender and caregiver status. We conducted several 2 (Gender: male vs female) x 2 (Caregiver status: caregiver vs non-caregiver) ANOVAs on the D score reflecting the differences between proportion of time per day in % spent on work, caregiving, housework, hobbies, and exercise before vs during the lockdown. The findings showed significant main effects of caregiver status on three outcomes, such that caregivers dedicated less time to work during the lockdown than before the pandemic, F(1, 220) = 15.82, p < .001, more to caregiving, F(1, 220) = 72.03, p < .001, and less to hobbies, F(1, 220) = 11.39, p = .001, compared to non-caregivers. A main effect of gender was observed for exercise, F(1, 220) = 9.09, p = .003, with men reporting that they spent less time on exercise during the lockdown vs before the pandemic compared to women. No significant interactions were discovered. These findings show that regardless of gender, caregiver status impacts the redistribution of time spent on different tasks during vs before the lockdown.
